# Exacerbation of financial burden of insulin and overall glucose‐lowing medications among uninsured population with diabetes

**DOI:** 10.1111/1753-0407.13360

**Published:** 2023-02-07

**Authors:** Yilu Lin, Hui Shao, Vivian Fonseca, Lizheng Shi

**Affiliations:** ^1^ Department of Health Policy and Management, School of Public Health and Tropical Medicine Tulane University New Orleans Louisiana United States; ^2^ Department of Pharmaceutical Outcomes and Policy, College of Pharmacy University of Florida Gainesville Florida United States; ^3^ Department of Medicine and Pharmacology, School of Medicine Tulane University New Orleans Louisiana United States

**Keywords:** financial burden, insulin, uninsured, 胰岛素, 无保险, 财务负担

## Abstract

**Background:**

Approximately 7.4 million Americans with diabetes used insulin. This study aimed to document the 10‐year trend of insulin and other glucose‐lowering medications expenditure among insured and uninsured populations and to examine the impact of insulin out‐of‐pocket (OOP) payment and insurance status on glucose‐lowering medication OOP expenditure.

**Methods:**

We extracted data from the Medical Expenditure Panel Survey (2009–2018) to document trends in the expenditure of insulin among people with diabetes. Total expenditures and OOP spending per person were documented on insulin and noninsulin glucose‐lowering medications among insured and uninsured populations. Multivariable regression was applied to assess the association of insulin OOP payment and insurance status on glucose‐lowering medication OOP expenditure.

**Results:**

Although insulin usage was stable over the decades, total insulin expenditure almost doubled per person per year after the Affordable Care Act (ACA) regardless of the insurance status. The OOP cost of insulin by the uninsured population increased from $1678 per person per year in the pre‐ACA period to $2800 per person per year in the post‐ACA period. After the ACA was enacted, the uninsured population had $403.96 and $143.64 more on OOP costs than the people with public and private insurance, respectively.

**Conclusion:**

For insured people, the rising financial burden of insulin was borne mainly by insurance. The uninsured population is bearing a heavy burden due to the high price of insulin. Policymakers should take action to reduce the insulin price and improve the transparency of the insulin pricing process.

## Graphical abstract

**Highlights**

- Approximately 7.4 million Americans with diabetes use one or more formulations of insulin and the price of insulin tripled from 2002 to 2013. There are limited studies to explore the impact of insulin price, particularly on the uninsured population.
- For insured people, there was little impact on the out‐of‐pocket (OOP) payment for patients with diabetes using insulin treatment during the insulin price rise. The burden was borne mainly by insurance.
- When the high insulin price issue came into the uninsured population, the financial burden became urgent because the consequences of rationing insulin are deadly. After the Affordable Care Act was enacted, the uninsured population had $403.96 and $143.64 more OOP payments than people with public and private insurance, respectively.

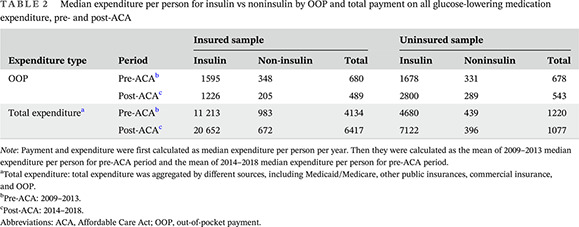

## 摘要

**背景:** 美国约有740万糖尿病患者使用胰岛素。本研究旨在记录有保险和无保险人群的胰岛素和其他降糖药物支出的10年趋势，并研究胰岛素自费支付和保险状态对降糖药物自费支出的影响。

**方法:** 我们从MEPS数据库(2009‐2018)中提取数据，记录糖尿病患者胰岛素支出的趋势。记录有保险和无保险人群中每个人在胰岛素和非胰岛素降糖药物上的总支出和自费支出。应用多元回归分析评估胰岛素自费支付和保险状态对降糖药物自费支出的影响。

**结果:** 尽管胰岛素的使用在过去十年中保持稳定，但平价医疗法案(ACA)实施后，无论保险状态如何，每个人每年的胰岛素支出几乎翻了一番。无保险人群的胰岛素自费成本从ACA实施前的每人每年1678美元增加到了ACA实施后的每人每年2800美元。ACA实施后，无保险人群的自费支出比有公共或私人保险的人群分别多出403.96美元和143.64美元。

**讨论:** 对于有保险的人来说，胰岛素不断增加的财务负担主要由保险承担。无保险人群由于胰岛素高价而承受了沉重的负担。政策制定者应采取行动降低胰岛素价格并改善胰岛素定价过程的透明度。

## 1 INTRODUCTION

Approximately 34 million Americans were estimated to have been diagnosed with diabetes in 2018.^1^ Among them, 7.4 million people with diabetes used at least one formulation of insulin.^1^ The population with type 1 diabetes mellitus (T1DM) would have to take insulin indefinitely because their bodies no longer make this hormone.^2^ Individuals with type 2 diabetes mellitus (T2DM) could manage the disease with a healthier lifestyle. Insulin therapy is recommended for a person with advanced T2DM when other medications have failed to maintain glycemic control.^3^

The rising prices of insulin products are anything but justifiable. One insulin Humalog (Lispro, 10 ml vial) was $21 in 1999, but cost $332 in 2019, reflecting a price increase of more than 1000%.^4^ The high price being attributed to the “high cost of development” does not apply to insulin because even the latest and most commonly used analog insulin products have been on the market for over 20 years or longer. This soaring price contributed to the total economic burden of diagnosed diabetes, which was estimated to be $327 billion in 2017.^5^, ^6^ One in three Medicare beneficiaries has diabetes, and 3.1 million Part D enrollees require insulin.^7^ Around 25% of the diabetes population covered by Medicare reported a reduction in the use of insulin owing to the rising cost.^8^ Beyond the economic burden, the rising insulin price also had clinical implications, especially for the most vulnerable subgroups. To lower the burden caused by insulin prices, people with diabetes rationed their insulin. They either skipped insulin injections or did not take enough to prolong each dose. There were people with diabetes who suffered severe complications (eg, diabetic ketoacidosis and end‐stage renal disease) and died owing to insulin rationing with the poor affordability of insulin.^9^ People even went to Canada to buy insulin because they could no longer afford insulin in the United States.^10^

The medication expenditure could be reimbursed partially by the insurance. Thus, the out‐of‐pocket (OOP) cost matters to every insulin user through the rising insulin price issue. OOP cost includes deductibles, coinsurance, and copayments for covered services plus all costs for services that are not covered.^11^ For uninsured and underinsured people, a large proportion of their medical expenses are OOP payments. Therefore, the vulnerable uninsured and underinsured population are more likely to live with restricted insulin access or even higher mortality risk, owing to the high cost of the insulin OOP payments.

The Affordable Care Act (ACA), which was enacted on March 23, 2010, addressed health insurance coverage, health care costs, and preventive care. In 2009, 17% of all adults with diabetes under age 65 were uninsured. After the ACA took effect, that number declined to 5%. Among low‐income adults with diabetes, 33% were uninsured before the ACA and 6% were uninsured after. In all, an additional 1.9 million people with diabetes—more than half of whom were low income—gained insurance coverage after the ACA.^12^ However, the act did not provide details about whether the expenses of prescription drugs including insulin syringes, insulin pumps, and infusion sets are covered and if these expenses would be applied before or after deductibles are met for those insured.^13^ Moreover, those who remained uninsured and underinsured after ACA, as the most vulnerable population, were still suffering from the high price of insulin.

Therefore, there is an urgent need to examine insulin expenditure and its impact on individuals, especially on the uninsured population. Prior studies looked into the insulin spending on either T1DM or T2DM in only cohort or elderly populations. Lipska et al discussed the OOP cost of insulin among the T2DM population from 2000 to 2010 using the Optum Labs Data Warehouse, private insurance claim data.^14^ There is another research group focused on insulin spending by the T1DM population.^15^ Researchers also investigated Medicare spending on insulin.^7^ Hua's team used the Medical Expenditure Panel Survey (MEPS) data from 2002 to 2013 and examined the price and expenditures of antihyperglycemic medication.^6^ After the ACA, the uninsured population is gaining more attention on the rising insulin price. This study aimed to document the 10‐year trend in insulin spending and other glucose‐lowering medications spending among the insured population and the uninsured population and to examine the impact of OOP expenditure for insulin and insurance status on the overall OOP cost for glucose‐lowering medication from 2009 to 2018.

## 2 METHODS

Ten‐year individual and prescription data from the MEPS 2009 to 2018 were extracted to describe and compare trends in the expenditure of insulin and insulin analogs among people with diabetes. The MEPS is a nationally representative household survey supplemented with data collected from pharmacies and other providers. Its Household Component (HC) collects demographic characteristics, health conditions, health status, use of medical services, charges and source of payments, access to care, satisfaction with care, health insurance coverage, income, and employment of each person interviewed. The overall response rate from MEPS‐HC in the study period ranges from 42.7% to 57.2%.^16^ To derive national estimates, MEPS data were weighted by the proportion of the population they represent. The longitudinal files derived from the respondents to the MEPS Panel and Full Year Consolidated Data File, Medical Conditions File, and Prescribed Medicines File were used for analysis.

Diabetes diagnosis was determined by the MEPS diabetes condition variable. If people were considered as treated for diabetes with medication, they must take glucose‐lowering medication and/or treat diabetes with insulin injections. Multum Lexicon Codes were used to identify glucose‐lowering medications. The non‐nsulin medications included metformin, thiazolidinediones, sulfonylureas, alpha‐glucosidase inhibitors, sodium‐glucose cotransporter‐2 (SGLT‐2) inhibitors, meglitinides, dipeptidyl peptidase‐4 (DPP‐4) inhibitors, amylin analogs, incretin mimetics, and antidiabetic combinations. OOP payment was indicated by “self/family” as a source of payment variable. Overall spending was aggregated by different sources, including Medicaid/Medicare, other public insurances (Veterans/CHAMPVA, Tricare, state and local government, other federal and other public), commercial insurances (private insurance, workers company insurance, and other private insurance), and OOP payments. Insurance status was grouped as insured (any private insurance or public insurance)) and uninsured. All expenditures were adjusted as the 2018 dollars using Consumer Price Index (CPI) for prescription drugs (CPI‐PMED).^17^ All the information from MEPS data was self‐reported and validated by the MEPS pharmacy sector.

The study documented the trend of the gross per‐person spending on insulin versus noninsulin medications and total glucose‐lowering medication expenditure on insured and uninsured populations. Descriptive statistics were used to describe individuals' characteristics including demographic characteristics, insurance status, and glucose‐lowering drug use in each year through the decade. Glucose‐lowering medication spending on the use of insulin vs noninsulin glucose‐lowering medications by OOP payment and total payment of insured and uninsured in pre‐ and post‐ACA period was presented. Annual median spending was also presented. Because of the disadvantage of being affected by any single value being too high or too low compared to the rest of the sample, we used median as a representative midpoint measurement instead of mean. Moreover, multivariate regression was conducted to explore the impact of insulin OOP payment and insurance status on overall glucose‐lowering medication OOP expenditure, controlling for demographic characteristics, treated diabetes, insurance types, quantity of insulin used among participants who used insulin, and ACA. ACA has an impact on insurance coverage and in turn, influences medication use. With a concern of collinearity with the ACA dummy variable (year 2013 and after), the calendar year variable was not included in the regression. The regression was clustered by year and variance estimation primary sampling unit provided by the MEPS data file to avoid serial correlation. The interaction term of insurance status and ACA was also included in the regressions. SAS “proc survey” was used for analysis as recommended by MEPS.^18^

## 3 RESULTS

A total of 13 696 participants were extracted from the MEPS data from 2009 to 2018. The medication information was recorded annually. The prevalence of insulin purchase has a small growth from 25% to 30%. Alpha‐glucosidase inhibitors, amylin analogs, and meglitinides were no longer recorded in the MEPS database since 2013, whereas SGLT‐2 started to take its place after its first agent in the class was approved in 2013. There was a reduction in the proportion of antidiabetic combinations, sulfonylureas, and thiazolidinediones use. The proportions of other medications including DPP‐4 inhibitors, incretin mimetics, and metformin increased over the years (Table 1).

**TABLE 1:** Glucose‐lowering medications of individuals with diabetes in MEPS, 2009–2018

Period	Measurement	Insulin	Alpha‐glucosidase inhibitors	Amylin analogs	Antidiabetic combinations	Dipeptidyl peptidase 4 inhibitors	Incretin mimetics	Meglitinides	Metformin	SGLT‐2 inhibitors	Sulfonylureas	Thiazolidinediones
2009	*N*	585	7	3	204	132	36	27	1097		728	326
	Weighted *N*	10 154 279	59 346	59 584	2 360 173	1 697 110	617 471	296 588	13 007 507		8 261 593	3 371 217
	%	25.46	0.15	0.15	5.92	4.26	1.55	0.74	32.61		20.71	8.45
2010	*N*	587	9	4	164	132	39	24	1053		668	252
	Weighted *N*	11 317 965	104 835	58 048	1 931 802	1 472 766	598 533	319 106	14 059 910		8 500 617	2 931 008
	%	27.41	0.25	0.14	4.68	3.57	1.45	0.77	34.05		20.59	7.10
2011	*N*	706	8	2	159	159	46	18	1250		714	207
	Weighted *N*	13 405 112	58 872	9349	1 853 624	1 848 915	714 732	164 260	15 194 909		8 670 611	2 307 894
	%	30.31	0.13	0.02	4.19	4.18	1.62	0.37	34.36		19.60	5.22
2012	*N*	730	12	3	178	196	43	21	1310		750	136
	Weighted *N*	12 813 380	106 348	41 911	1 753 823	2 127 144	648 374	217 240	14 475 587		8 396 786	1 594 361
	%	30.38	0.25	0.10	4.16	5.04	1.54	0.52	34.32		19.91	3.78
2013	*N*	758			167	196	65		1366		708	91
	Weighted *N*	12 348 071			1 394 241	2 028 807	994 253		15 441 175		8 316 737	1 076 205
	%	29.68			3.35	4.88	2.39		37.12		19.99	2.59
2014	*N*	773			157	198	70		1377	44	654	106
	Weighted *N*	16 115 118			1 973 929	1 949 723	1 149 108		17 624 692	488 226	8 763 048	1 381 002
	%	32.59			3.99	3.94	2.32		35.65	0.99	17.72	2.79
2015	*N*	777			180	217	87		1405	88	706	100
	Weighted *N*	13 551 525			1 939 384	2 522 495	1 387 565		16 756 480	1 202 470	8 475 283	1 062 595
	%	28.90			4.14	5.38	2.96		35.73	2.56	18.07	2.27
2016	*N*	797			175	253	108		1451	101	675	101
	Weighted *N*	14 222 474			1 592 458	3 014 126	1 340 502		18 039 777	1 409 845	8 387 497	1 221 826
	%	28.89			3.23	6.12	2.72		36.65	2.86	17.04	2.48
2017	*N*	790			136	259	153		1379	119	633	90
	Weighted *N*	14 433 186			1 724 238	2 743 806	1 818 651		17 616 713	1 682 094	8 199 396	1 084 780
	%	29.27			3.50	5.57	3.69		35.73	3.41	16.63	2.20
2018	*N*	769			143	263	192		1489	134	599	102
	Weighted *N*	15 301 094			1 793 750	3 042 702	2 706 130		18 793 944	1 720 856	7 993 954	1 287 081
	%	29.07			3.41	5.78	5.14		35.70	3.27	15.19	2.45

Abbreviations: MEPS, Medical Expenditure Panel Survey; SGLT2, sodium‐glucose cotransporter‐2.

By comparing the median OOP expenditure and overall medication expenditure between the insured population and uninsured population, the insulin OOP cost of individuals with insurance even decreased by approximately $300, and their overall insulin expenditure doubled from $11 213 to $20 652 because of the implementation of ACA and Medicaid expansion. In contrast, for the uninsured population, the insulin OOP payment had an overall increasing trend from $1678 to $2800. The noninsulin OOP payments and the overall OOP payment, however, decreased after the ACA for uninsured population. These results suggested that the increased insulin expenditure carried a larger impact on the uninsured population because the main payment method for these people was OOP. When looking into the noninsulin medication costs, noninsulin OOP expenditure and noninsulin total expenditure decreased regardless of insurance status (Table 2). The medication OOP overall (noninsulin OOP payment and insulin OOP payment together) decreased by approximately 33% (from $818 to $546) and 47% (from $804 to $429) for the insured population and uninsured population, respectively (Table A1). It indicated that the decrease in glucose‐lowering medication OOP costs was contributed by noninsulin OOP payment because there was a large drop in noninsulin OOP. Moreover, though there was little variation in insulin OOP payment for the individuals with insurance coverage, the insulin OOP payment had a high increase from the uninsured population. Jonckheere–Terpstra test was conducted and indicated the trend was statistically significant.

**TABLE 2:** Median expenditure per person for insulin vs noninsulin by OOP and total payment on all glucose‐lowering medication expenditure, pre‐ and post‐ACA

Expenditure type	Period	Insured sample			Uninsured sample		
		Insulin	Non‐insulin	Total	Insulin	Noninsulin	Total
OOP	Pre‐ACA^b^	1595	348	680	1678	331	678
	Post‐ACA^c^	1226	205	489	2800	289	543
Total expenditure^a^	Pre‐ACA^b^	11 213	983	4134	4680	439	1220
	Post‐ACA^c^	20 652	672	6417	7122	396	1077

*Note*: Payment and expenditure were first calculated as median expenditure per person per year. Then they were calculated as the mean of 2009–2013 median expenditure per person for pre‐ACA period and the mean of 2014–2018 median expenditure per person for pre‐ACA period.

^a^ Total expenditure: total expenditure was aggregated by different sources, including Medicaid/Medicare, other public insurances, commercial insurance, and OOP.

^b^ Pre‐ACA: 2009–2013.

^c^ Post‐ACA: 2014–2018.

Abbreviations: ACA, Affordable Care Act; OOP, out‐of‐pocket payment.

In the regression analysis, age was transformed into a categorical variable by a cutoff point of 65 years old based on the Medicare coverage age limit. The regression explored the impact of insulin OOP payment and insurance status on glucose‐lowering medication OOP expenditure. Compared with White people, the Asian population spent $246.37 more on glucose‐lowering medication, whereas the Black and Hispanic populations spent $190.95 and $238.78 less on glucose‐lowering drugs OOP cost, respectively. The implementation of ACA increased the overall glucose‐lowering medications expenditure by $266.30. After the ACA, people with public and private insurance had $403.96 and $143.64 lower OOP payments than the uninsured population, respectively. These statistics indicated that the ACA did alleviate the OOP cost for the insured population; however, for the uninsured population, the impact of the ACA was exacerbated (Table 3).

**TABLE 3:** Estimated regression coefficients of factors associated with OOP expenditure

Parameter	Estimate	Standard error	*p* Value
Age ≥ 65 years	ref		
Age < 65 years	60.59	79.77	.4476
Male	ref		
Female	−75.92	69.05	.2716
Race White	ref		
Race Asian	246.37	463.93	.5954
Race Black	−190.95	76.76	.0129
Race Hispanic	−238.78	77.98	.0022
Race Other	−179.28	129.24	.1655
Treated diabetes with diet	ref		
Treated diabetes with medication	81.37	92.85	.3809
Insulin quantity	−1.33	0.66	.0433
Uninsured	ref		
Any private insurance	91.33	151.99	.5480
Public insurance only	119.45	176.47	.4985
ACA	266.30	245.45	.2781
Uninsured*ACA	ref		
Any private insurance*ACA	−143.64	258.45	.5791
Public insurance only*ACA	−403.96	273.30	.1395
Insulin OOP	1.01	0.00	<.0001

Abbreviations: ACA, Affordable Care Act; OOP, out‐of‐pocket payment.

## 4 DISCUSSION

In this study, we observed an increasing trend in insulin expenditure. For people with insurance, insulin OOP cost was not greatly affected because health insurance payments covered the gap generated by the rapidly increasing insulin expenditure. From the uninsured population side, however, the OOP payment of this essential medication increased 1.7 times along with the increased insulin price. The ACA and its provisions like Medicaid Expansion and Marketplace subsidies did remove a large proportion of the population from uninsured status. The population that remained uninsured, however, was the most vulnerable group that was sensitive to the disease's financial burden.^19^ The magnitude of estimated coefficients in the regression analysis indicated that health insurance, public or private, played an important role in the overall glucose‐lowering medication OOP expenditure. But for the uninsured population, high OOP expenditure caused by the high price of insulin treatment posed a large barrier that may decrease their adherence and threaten their lives.

Our findings are consistent with a study by the Centers for Disease Control and Prevention that used the private insurance MarketScan Claims database showing the increase in insulin spending was covered by payers.^20^ For example, the policy on closing the Medicare Part D coverage gap traded off the impact of higher insulin prices, which kept the OOP cost in check.^21^ Nevertheless, insurance companies could use their buyer power to bargain and receive discounts and rebates from the pharmacy companies on insulins in return for their formulary status.^22^ Our study suggested that the rising insulin price became a big problem for uninsured people who would have to pay full price for insulin prescriptions, whereas people with insurance could be better shielded by the insurance coverage plan. It was always the uninsured population who were more vulnerable. One study confirmed our conclusion for the insured people that used claims data of privately insured enrollees, which indicated high reimbursement proportion.^14^ A recent study found that 14.1% of insulin users spent 40% of their postsubsistence family income on insulin alone over 1 year, representing almost 1.2 million people. Approximately two thirds of people who experienced this high spending on insulin were Medicare beneficiaries.^23^ Insulin expenditure is a great burden even for the insured population with government assistance. How this high OOP payment affects the uninsured population is imaginable. It was appalling to see a six‐times increase in the insulin OOP cost for the uninsured in our study. Moreover, the national health reform reduced the burden for people with preexisting conditions and increased insurance coverage; however, those unable to pay the insurance premium still existed and these people are the most vulnerable population. Therefore, previous studies and our study raised the concern about the uninsured/underinsured population because they had the high OOP cost of insulin without a third‐party payer. Considering the great impact on uninsured low‐income, minority populations who need insulin treatment and the unreasonably high price for this life‐saving medication, the disparities and inequities in insulin access need to be addressed through multifaceted national actions.

Both government and industry are making efforts to satisfy the need for insulin and to decrease the price of insulin in many ways. Congress and state governments are discussing action to relieve the burden of rising insulin prices.^24^, ^25^, ^26^, ^27^ The Centers for Medicare & Medicaid Services is offering lower insulin costs through enhanced Part D prescription drug plans will have access to a broad set of insulins at a maximum $35 copay for a month's supply, from the beginning of the year through the Part D coverage gap.^28^ Pharmaceutical companies offer support programs to defray the costs for those who are experiencing high OOP costs, including coupons, free samples, and patient assistance programs (PAPs).^29^, ^30^, ^31^, ^32^ However, for the uninsured population, the accessibility of insulin was still limited because of the high price. Finding information about these PAPs can be difficult, and PAPs often have complicated income, insurance, and prescription requirements even with the information collected.

Our study had several limitations. We used patient‐reported data from a national survey with a response rate ranging from 42.7% to 57.2%. The survey participants have the possibility of faulty recall or other ascertainment bias and volunteer bias. We did observe certain outliers in Table 2, although these outliers did not affect the overall increasing/decreasing trend. One explanation of this might be the different sample: MEPS data were not able to follow up with households for a very long period, which may change the characteristics largely in a certain year and caused the outliers.^33^ Furthermore, the physical condition and diabetes duration were not taken into consideration as control variables in our study because self‐reports may not perfectly conform to diagnoses made by physicians. Insulin use adherence and appropriateness are important factors; however, the information is not contained in the MEPS data. Our study controlled only for insulin quantity, which may not get a full picture of the research question. Usage of free medication samples, insurance premiums, and the amount of tax paid were not included in our analyses because of the data availability. However, the payment amount was validated with pharmacy records for prescription drugs., which ensured our data quality and the credibility of the results.

## 5 CONCLUSION

Our study found that the price of insulin and its analogs increased. For the insured cohort, the financial burden of this rapid price change was covered by insurance. The insulin OOP payment among insured individuals with diabetes was stable. When the high insulin price issue came to the uninsured population, the financial burden became urgent because the consequences of rationing insulin are significant. The irrational increase in insulin price, however, remained an unresolved issue after ACA. It is imperative to slow down the increasing expenditure trend by reducing insulin costs. Additionally, more supportive policies should be implemented for the uninsured diabetes population to get enough necessary insulin usage.

## ACKNOWLEGEMENTS

None.

## FUNDING STATEMENT

No funding.

## DISCLOSURE

No conflict of interest.

## ETHICS APPROVAL STATEMENT

Not applicable.

## PATIENT CONSENT STATEMENT

Not applicable.

## PERMISSION TO REPRODUCE MATERIAL FROM OTHER SOURCES

Not applicable.

## CLINICAL TRIAL REGISTRATION

Not applicable.

## Data Availability

MEPS is a publicly available survey data source.

## APPENDIX A

**TABLE A1:** Median expenditure per person for insulin vs noninsulin by OOP and total payment on all glucose‐lowering medication expenditure, 2009–2018

Expenditure type		Insured sample			Uninsured sample		
	Year	Insulin	Noninsulin	Total	Insulin	Noninsulin	Total
OOP	2009	1509.27	418.72	817.91	1149.49	441.7	804.3
	2010	1439.3	388.67	752.68	1598.12	291.63	459.18
	2011	1455.42	361.49	703.15	1513.36	337.39	745.24
	2012	2107.2	313.77	582.63	3019.67	328.53	725.35
	2013	1464.46	256.89	541.24	1110	253.88	657.18
	2014	1456.74	242.11	525.14	691.05	222.31	542.96
	2015	1202.2	186.5	470.96	2196.76	255.98	433.52
	2016	1074.79	210.8	467.99	2115.49	396.5	611.03
	2017	946.33	185.39	434.12	2472.59	383.13	698.96
	2018	1448.91	201.24	545.59	6522.29	186.93	428.92
Total expenditure^a^	2009	10 165	1350.37	4665.91	3215.53	586.9	1369.37
	2010	10 439	1030.66	4235.08	4928.52	438.94	976.74
	2011	9371.14	877.99	3744.03	2418.26	429.02	956.49
	2012	14 449	847.37	4201.54	8451.94	375.29	1426.28
	2013	11 642	810.94	3823.36	4383.61	362.94	1370.67
	2014	18 980	683.56	4375.36	3559.87	442.16	822.23
	2015	19 275	745.53	5838.97	6692.56	439.17	882.88
	2016	19 886	662.54	5544	4202.47	510.11	1725.61
	2017	21 440	681.85	7911.38	13 396	407.8	1121.39
	2018	23 681	587.62	8416.4	7756.94	182.22	834.07

^a^ Total expenditure: total expenditure was aggregated by different sources, including Medicaid/Medicare, other public insurances, commercial insurance, and OOP.

Abbreviation: OOP, out‐of‐pocket payment.
